# An Exploratory Genomic and Transcriptomic Analysis Between *Choloepus didactylus* and *Homo sapiens*

**DOI:** 10.3390/genes16030272

**Published:** 2025-02-25

**Authors:** Ariella Baran, Antony Ibrahim, Yuka Nakano, Hideyuki Aoshima, Takeshi Ozeki, Iri Sato-Baran, David D. Ordinario

**Affiliations:** 1 Miss Porter’s School, Farmington, CT 06032, USA; ariella_baran@genesis-healthcare.jp; 2 Donald Bren School of Information and Computer Sciences, University of California, Irvine, Irvine, CA 92697, USA; antony_ibrahim@genesis-healthcare.jp; 3 Genesis Institute of Genetic Research, Genesis Healthcare Co., Yebisu Garden Place Tower 26F, 4-20-3 Ebisu, Shibuya-ku, Tokyo 150-0013, Japan; yuka_nakano@genesis-healthcare.jp (Y.N.); hideyuki_aoshima@genesis-healthcare.jp (H.A.); takeshi_ozeki@genesis-healthcare.jp (T.O.); sato@genesis-healthcare.jp (I.S.-B.)

**Keywords:** *Choloepus didactylus*, sloth, transcriptomic analysis, differentially expressed genes (DEGs), metabolism, cancer, whole genome sequencing (WGS)

## Abstract

*Background/Objectives:* Sloths, a group of xenarthran mammals currently comprising six recognized distinct species, have been the focus of much physiological animal research due to their extremely slow metabolisms, deliberate movements, and their status as a species relatively unchanged for over 26 million years. However, despite all the effort aimed at understanding these unique characteristics, the sloth genome remains largely unexplored. Due to the link between genetics and observed traits, such an investigation could potentially lead to insights regarding the genetic basis of unique sloth behaviors and characteristics, such as slow movement, low metabolism, and longevity. *Methods:* In this exploratory investigation, we performed whole genomic and transcriptomic analysis of a female *Choloepus didactylus* (Linnaeus’s Two-Toed Sloth). Through whole genome sequencing (WGS), the genetic overlap between female two-toed sloths and female humans was estimated in line with evolutionary biology. *Results:* Transcriptome analysis of peripheral blood mononuclear cells (PBMCs) showed significant differences between gene expression levels in two-toed sloths and humans related to metabolism, body temperature control, cell cycle regulation, telomere maintenance, circadian rhythm regulation, and cancer prevention. *Conclusions:* The discovered differences imply a relationship to the low metabolisms, slow movements, and longevity displayed by sloths. Future exploratory research will include additional testing to determine if these findings are universal among all recognized sloth species, as well as to address the relationship between specific gene and protein functions and observed traits.

## 1. Introduction

The members of the superclade Xenarthra, an ancient order native to the Americas which includes sloths, anteaters, and armadillos, represents the earliest derived basal clade of placental mammals and features the lowest metabolism among all mammals [1,2,3]. Sloths can be categorized into six distinct species divided into two families: *Choloepodidae* and *Bradypodidae*. Despite a significant divergence between the two families ~26.9 million years ago that would imply the subsequent development of different traits [4,5], each of these six species share unique physiologies, behaviors, and physical features including slow metabolic rates, long sleeping durations, heterothermia, low incidences of cancer, and extremely slow cell proliferation rates [6]. These unique physiological characteristics have been hypothesized to aid sloth survival by enabling them to adapt and take advantage of their environment while making them less noticeable to and keeping them out of reach from predators [7,8]. In spite of all this, no comprehensive genetic exploration between sloths and humans has been conducted. The benefits of such a study could potentially lead to further research on protein functions specific to the unique phenotype and survival abilities of a group of mammals that have lived for over 26 million years despite their disadvantaged physiology against both predators and the natural environment.

In this study, we performed a genomic and transcriptomic analysis of *C. didactylus* (Linnaeus’s two-toed sloth, hereafter referred to as ‘two-toed sloth’). First, we obtained whole genome sequence (WGS) and RNA-Seq data using samples obtained from a two-toed sloth. Next, we compared the two-toed sloth WGS data with *H. sapiens* (human) data. Finally, we performed a differential gene expression (DEG) analysis between the two-toed sloth and humans. Based on our analysis, we were able to gain insight into the amount of genetic overlap between two-toed sloths and humans, as well as compare the expression levels of genes present in both. These findings demonstrate the first known direct genomic and transcriptomic comparison between two-toed sloths and humans and open a path for future investigations into specific gene and protein functions tied to the unique characteristics of the sloth species.

## 2. Materials and Methods

### 2.1. Two-Toed Sloth and Human Volunteer Profiles

The two-toed sloth used for this study was a two-year-old female Linnaeus’s two-toed sloth weighing 6.82 kg. The two-toed sloth was born in Guyana and has been privately owned in Tokyo, Japan, by the lead author from approximately 2 months old. Human volunteer ‘A’ was a Japanese female in her mid-thirties, while human volunteer ‘B’ was a Japanese female in her mid-fifties. Only two human volunteers participated due to this study being a pilot intended to make an initial genetic comparison between sloths and humans and to develop a standardized methodology for performing the comparison. All procedures involving the two-toed sloth were performed in line with the Japanese Act on Welfare and Management of Animals [9]. Ethical approval and informed consent were obtained from all human participants.

### 2.2. Collection and Processing of Blood Samples for Genetic Sequencing

Two-toed sloth blood samples were collected by a licensed veterinarian (Pet Medical Japan, Asahi-shi, Chiba, Japan) familiar with exotic and zoo animals. 2.4 mL of blood was collected by percutaneous femoral vein puncture with BD Microtainer EDTA Vacutainer tubes (Becton Dickinson and Company, Franklin Lakes, NJ, USA). Human blood samples were collected from two healthy adult female volunteers by a registered nurse (Souseikai Clinic, Tokyo, Japan) via venipuncture using TERUMO Venoject II EDTA Vacutainer tubes (TERUMO, Tokyo, Japan). Three 5 mL vials were drawn from each person to obtain a sufficient concentration of DNA. Immediately after each collection, all blood samples (both sloth and human) were placed in Styrofoam containers with icepacks to maintain a temperature below 22 °C during transport. Immediately after arrival at the lab, blood samples were frozen at −80 °C to preserve the DNA for later analysis. Peripheral blood mononuclear cells (PBMCs) for both the two-toed sloth and the human samples were fractionated by density gradient centrifugation using Lymphoprep^TM^ (Serumwerk Bernburg AG, Bernburg, Germany).

### 2.3. RNA Extraction, Library Setup, and Sequencing

Total RNA for the mononuclear cells was isolated using ISOGEN (NIPPON GENE, Tokyo, Japan) based on the phenol–chloroform extraction method. RNA libraries were prepared using an MGIEasy RNA Directional Library Prep Set (MGI Tech Co., Ltd., Shenzhen, China). Both libraries were sequenced using an MGI DNBSEQ-T7 sequencer (MGI Tech Co., Ltd., Shenzhen, China). The average alignment coverage over WGS was 2.46 for the sloth, 0.62 for human volunteer ‘A’, and 1.93 for human volunteer ‘B’.

### 2.4. Preprocessing of RNA-Seq Data

The two-toed sloth and human RNA-Seq data was run through DRAGEN (Illumina, San Diego, CA, USA) in order to generate read count data, which was generated in .sf format. Python 3.12.4 (Anaconda distribution 2024.06-1) was used to extract the count data and load them into a Pandas DataFrame using the Pandas library. Once imported, the data was filtered to remove samples with no read count, samples with total read counts of less than 10, and samples with invalid names. All genes were cross-referenced with Ensembl through the Ensembl REST API (GrCh38) to ensure validity.

### 2.5. Determination of Differentially Expressed Genes (DEGs)

DEG analysis was performed using Python 3.12.4 (Rpy2 3.5.13) and R 4.4.1. First, normalization was performed according to DESeq2′s median of ratios method. After a second filtering process to remove low or no read count samples and extreme outliers (>3 standard deviations from the mean), potential DEGs were determined using DESeq2 1.46. The two-toed sloth samples were set as the baseline, and separate comparisons were performed for each individual human sample. Finally, a list of DEGs was determined by excluding all genes with a log2 fold change of less than 1 and/or an adjusted *p*-value greater than 0.1.

### 2.6. Two-Toed Sloth Genome Assembly

Based on previously published reads (*C. didactylus* genome, mChoDid1, sequence data Accession: PRJNA1008600 s (GenBank: AY960980.1)), the two-toed sloth genome was assembled into contigs.

## 3. Results

**Figure 1:**
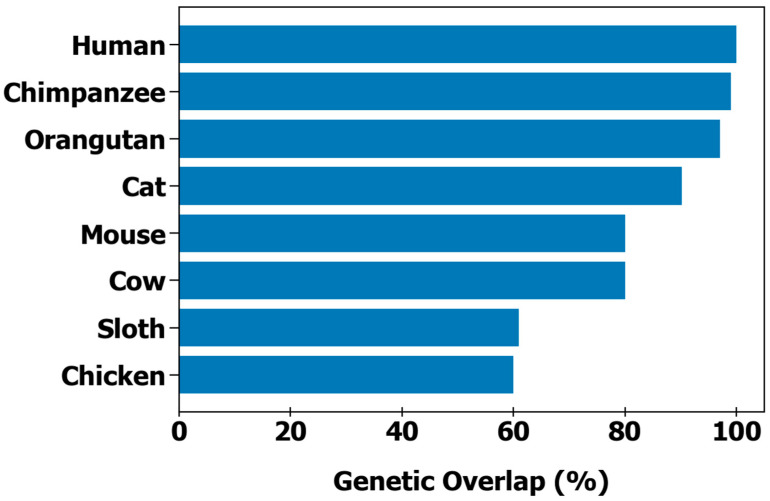
Estimated genetic overlap between humans (*H. sapiens*) and various animal species. Based on WGS data, Linnaeus’s two-toed sloth has ~61% overlap with humans, placing it between chickens (60%) and cows (80%). Genetic overlap is defined as the number of shared orthologs between two species.

We began our studies by collecting and analyzing WGS data for both the two-toed sloth and humans. The two-toed sloth WGS data was mapped against the female human genome sequence, and both were compared (Table 1, Tables S1 and S2). The two-toed sloth genome contained 28 chromosomes and had a length of 3,130,174,040 base pairs, while the human female genome contained 23 chromosomes and had a length of 3,088,286,401 base pairs. Furthermore, the shared orthologs for the two-toed sloth mapped against humans varied by chromosome, from only 2 in Chromosome Y to 1525 for Chromosome 1. Regarding the length of each chromosome, the two-toed sloth had a longer read length for chromosomes 1–4, 8, 19, 20, and 21, while the read length for the remaining chromosomes was longer in humans. When mapped chromosome to chromosome, certain areas of each two-toed sloth chromosome corresponded to areas on different human chromosomes. For example, two-toed sloth chromosome 1 corresponded to areas of human chromosomes 3 and 21. A full list of corresponding areas is displayed in the Supplementary Materials (Tables S1 and S2). The number of shared orthologs between the human and two-toed sloth genomes show ~61% genetic similarity (defined here as the number of shared orthologs), which suggests that the amount of genetic overlap with the two-toed sloth falls between chickens and cows (Figure 1) [10,11,12,13,14,15].

**Figure 2:**
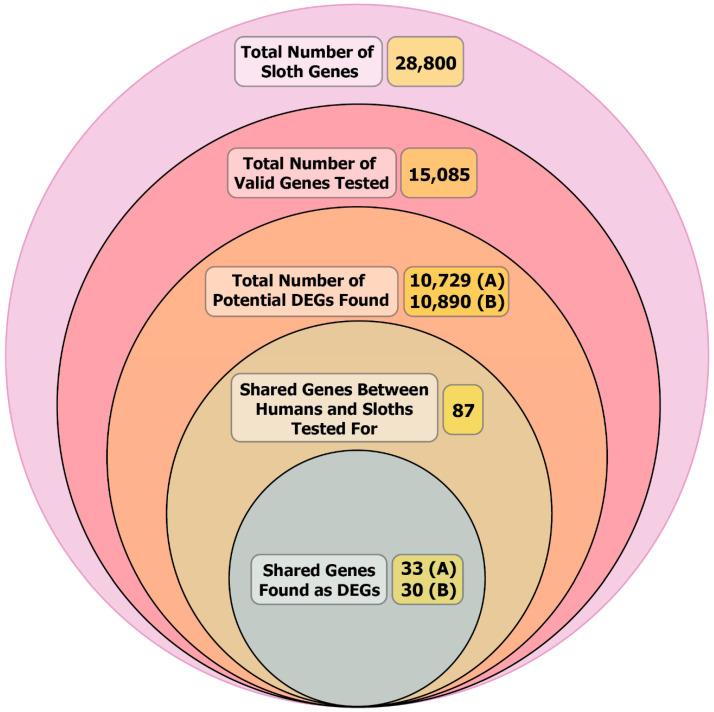
Onion diagram showing the number of two-toed sloth genes matching various conditions. A total of 28,800 two-toed sloth genes were identified. After filtering for null values, low or no read counts, and invalid names, the number of valid genes tested was 15,085. A total of 10,729 potential DEGs were identified between the two-toed sloth and human volunteer ‘A’, while 10,890 were identified between the two-toed sloth and human volunteer ‘B’. Based on a list of 87 shared genes related to specific two-toed sloth traits, the final number of shared genes found as DEGs came to 33 based on the comparison with human volunteer ‘A’ and 30 for the comparison with human volunteer ‘B’.

**Figure 3:**
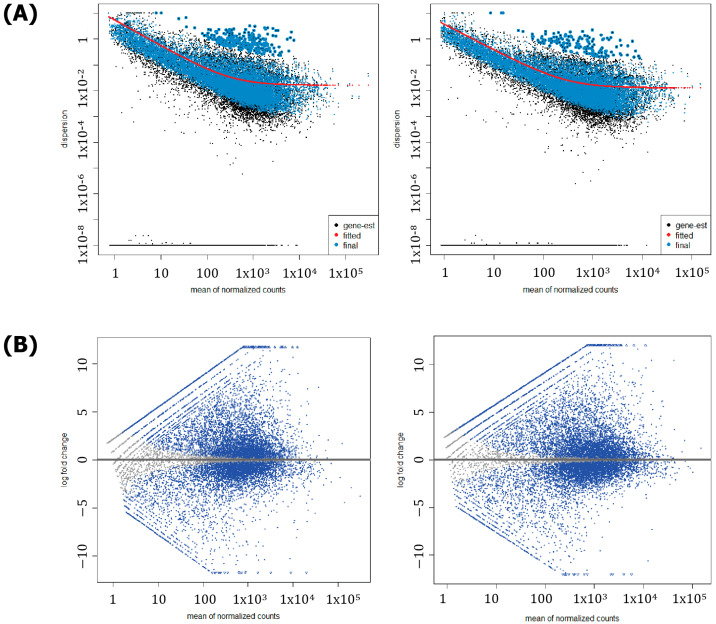
(**A**) Dispersion plots for the DEG analysis between the two-toed sloth and human volunteer ‘A’ (**left**) and human volunteer ‘B’ (**right**). An inverse relationship can be observed between mean counts and dispersion. (**B**) MA plots for the DEG analysis between the two-toed sloth and human volunteer ‘A’ (**left**) and human volunteer ‘B’ (**right**). Blue dots indicate genes that are likely to be differentially expressed, while gray dots indicate genes that are unlikely to be differentially expressed.

Next, a transcriptome analysis was performed between two-toed sloths and humans to elucidate the genetic basis of their marked biochemical, physiological, and behavioral characteristics. Specifically, RNA-Seq was performed on both species’ data to estimate differences in gene expression. The resulting data was then processed using standard bioinformatics tools. Afterwards, a DEG analysis was performed, where the two-toed sloth was compared to both humans separately. From a total of 28,800 (23,626 protein-coding) two-toed sloth genes, 15,085 genes were tested after removing genes with no read counts, null values, or invalid names (Figure 2). Based on this number, 10,729 potential DEGs were identified between the two-toed sloth and human volunteer ‘A’, while 10,890 were identified between the two-toed sloth and human volunteer ‘B’. The slight differences in expression may be attributable to the age difference between both human volunteers [16]. To visualize the potential DEGs, dispersion and MA plots for both analyses are shown in Figure 3A,B while heatmaps are shown in the Supplementary Materials (Figures S1 and S2).

**Figure 4:**
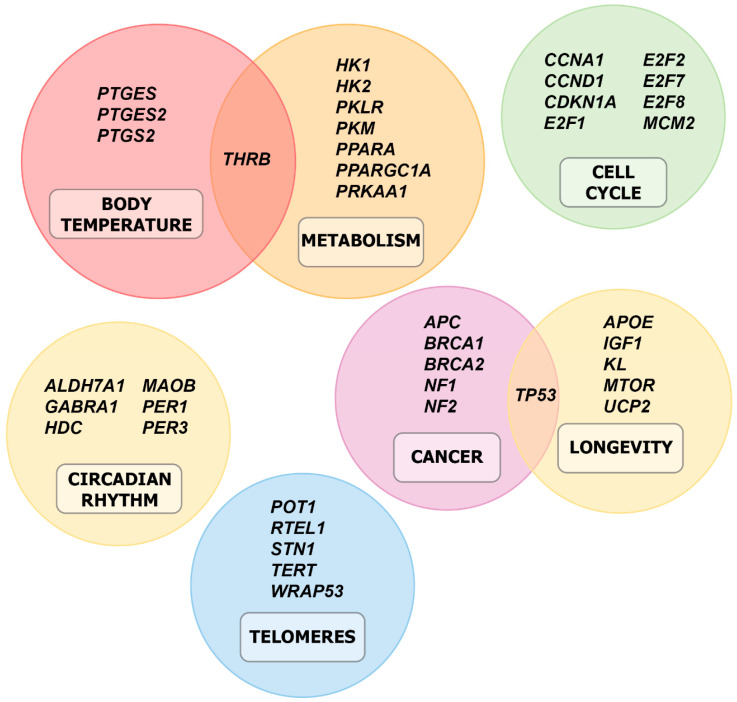
Cluster diagram showing the relationships and groupings of identified DEGs. Statistically significant (fold change > 1, *p* < 0.1) DEGs were grouped into the following 7 categories: body temperature, metabolism, cell cycle regulation, circadian rhythm, cancer, longevity, and telomere maintenance.

As identified in the orthologs between two-toed sloth and human genes, the list of potential DEGs was screened for 87 genes associated with the unique physiological characteristics and abilities of two-toed sloths (Figure 2). After screening, a total of 33 relevant DEGs were found between the two-toed sloth and human volunteer ‘A’, while 30 were identified between the two-toed sloth and human volunteer ‘B’. The results of the screening are shown in Figure 4, where the genes are clustered into a number of categories including body temperature, metabolism, cell cycle, circadian rhythm, telomere maintenance, cancer, and longevity.

Finally, observational data related to two-toed sloth food intake and waste excretion was collected (Table 2). The two-toed sloth’s average calorie intake was 228.8 kcal/day compared to that of the daily intake of an average Japanese female (~2000 calories) [17]. The two-toed sloth’s diet comprised of a combination of leafy greens, bananas, blueberries, carrots, cucumbers, sweet potato, and bread. The average feces weight was 33.6 g per day while the average urine volume was 81.8 mL per day.

## 4. Discussion

Based on the list of shared DEGs, genes were clustered based on similarities and shared functionalities that were biologically relevant to two-toed sloths. Notable categories included metabolism, cell cycle regulation, circadian rhythm, telomere maintenance, and cancer. Taken together with known information in the scientific literature, as well as collected observational data, several connections were made to the characteristics and behavior of the two-toed sloth.

**Figure 5:**
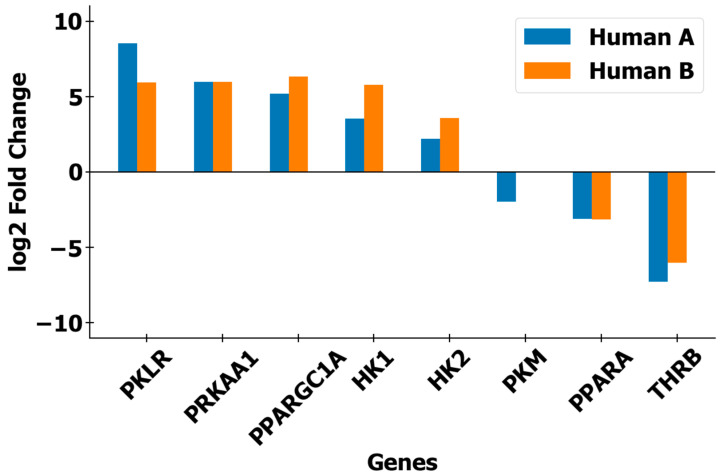
Representative bar graph showing the fold changes for DEGs associated with metabolism. For five of eight DEGs, there was higher expression in the two-toed sloth. For the remaining three DEGs, there was lower expression in the two-toed sloth. The five upregulated genes are linked to glycolysis, whereas the three downregulated genes are linked to lipid metabolism. For *PKM*, downregulation was observed when the sloth was compared with human volunteer ‘A’, but no significant downregulation was observed for human volunteer ‘B’. DEG fold changes for the other six identified categories can be found in the Supplementary Materials (Figures S3–S9).

For metabolism, genes related to glycolysis enzymes (*PKLR*, *HK1*, *HK2*, *PRKAA1*, *PPARGC1A*, *PPARA*) [18,19,20] were found to be upregulated relative to humans (Table 3, Figure 5). As two-toed sloths are herbivorous there is a heavy reliance on glycolytic pathways, and upregulated enzyme expression would potentially maximize effective utilization of limited available calories from plant sources in the sloth’s natural habitat. Previous reports [21], as well as the observed low daily caloric intake and food types (Table 2), further support this theory. Additionally, genes related to lipid metabolism (*PPARA*, *THRB*) were found to be downregulated relative to humans (Table 3, Figure 5). This could be related to the fact that, as herbivores, two-toed sloths do not need to rely as much on lipid-containing foods. All of these factors may be related to the slow, deliberate movements of two-toed sloths and complement existing environmental, behavioral, and evolutionary-based explanations, such as low-calorie plant-based diets, predator avoidance behavior, and adaptation for survival in rainforests [7,8].

For body temperature, the genes *PTGES2* and *THRB* were found to be downregulated relative to humans (Table S3, Figure S3). These genes are responsible for regulating body temperature [22,23], which is naturally low in two-toed sloths. This could help explain why two-toed sloths need to live in a tropical climate and rely on heat from the sun [24], why they primarily rely on behavioral methods to regulate their body temperature [25], and why they need to be metabolically efficient with the food resources available to them [26].

For cell cycle regulation and cancer, the genes *CCND1* and *E2F7* (cell cycle regulation, Table S3, Figure S4) and *BRCA2*, *NF2*, *BRCA1*, and *NF1* (cancer, Table S3, Figure S5) were found to be upregulated relative to humans. *CCND1* encodes Cyclin D1, which, when deregulated, becomes an oncogene and is recognized as a driver of solid tumors and hemopathies [27]. *E2F7* is related to the DNA repair process [28]. As for the remaining genes, they are representative tumor suppression genes [29]. Taken together, it is possible that the high expression levels of all these genes lead to a low incidence of cancer in two-toed sloths [30]. This apparent increased cancer resistance may have been inherited from the extinct large-bodied ancestors of modern sloths, where the anti-cancer genes may have been one solution to Peto’s paradox [30,31].

For circadian rhythms, the genes *PER1*, *PER3*, *GABRA1*, and *ALDH7A1* were found to be upregulated relative to humans (Table S3, Figure S6). *PER1* and *PER3* are core genes of the circadian clock, and any changes to their regulation status would have direct effects on circadian rhythms [32]. It is possible that the high expression of *GABRA1* (the GABA receptor) [33,34] explains the high activity of the GABA pathway, resulting in the observed long sleep durations in two-toed sloths. Additionally, *ALDH7A1* is known to be involved in the degradation of histamine and its metabolites [35], and has also been reported to promote sleep by inhibiting some part on neurons [36]. Although the *CLOCK*, *PER2*, *CRY1*, and *CRY2* genes were detected, their fold change and *p*-values did not indicate a statistically significant change in expression (Table S3, Figure S7). In short, all the genes identified could play a role in long two-toed sloth sleep times through the promotion or inhibition of processes associated with waking. The results also provide more evidence for sloths’ observed predator avoidance behavior through the timing of their sleep periods [37].

For telomere maintenance, the genes *RTEL1*, *WRAP53*, and *POT1* were found to be upregulated relative to humans (Table S3, Figure S8) [38]. Two-toed sloth cells are known to have extremely long doubling times [30]. Due to this, it may not be easy to replace cells in two-toed sloth tissue due to the slow proliferation. Thus, for two-toed sloths, cell protection may be of paramount importance. Additionally, the extremely long cell doubling time may reduce the total lifetime number of divisions. This would potentially keep the cells relatively young, resulting in the strong upregulation of genes related to telomere maintenance [39,40,41]. In short, differences in two-toed sloth gene expression regulation may help explain how they are able to live up to 30 years despite many of their inherent limitations compared to other animals.

For longevity, the genes *UCP2*, *MTOR*, *TP53*, *IGF1*, and *APOE* were found to be upregulated relative to humans, while *KL* was found to be downregulated relative to humans (Table S3, Figure S9) [42,43,44,45,46,47]. While *UCP2* is upregulated in sloths relative to both human volunteers, *MTOR* and *TP53* are only upregulated relative to human volunteer ‘A’, whereas *IGF1* and *APOE* are only upregulated relative to human volunteer ‘B’. Although the observed results for *UCP2* and *IGF1* are expected due to expression levels decreasing with age, the other observed results may be a result of individual variation among the human participants [48,49,50,51,52,53,54]. Nevertheless, despite these potential individual variations, it is apparent that the expression level for these genes is overall higher in the sloth, matching previous reports of high cancer resistance among xenarthrans [30]. In light of the link between energy expenditure and lifespan [55], it is plausible that the upregulation of almost all identified longevity-related genes in the two-toed sloth is a byproduct of their ability to efficiently use the limited energy accessible to them due to their low-calorie plant-based diet and need to avoid predators by limiting movement [7,8]. Together with the observed metabolism-related genes, these genes may play a role in the sloth aging process through controlling oxidative stress and damage [56]. On a broader level, these genes and the mechanisms underlying them may also play a role as natural sources of longevity in long-lived mammals of similar evolutionary lineage [57].

The observed unique DEG clusters and expression profiles can assist current and future sloth conservation efforts. Based on the identified metabolism and body temperature-related genes, sloths are highly adapted and optimized to their native habitat. This implies that, unless they can be placed in an environment that closely replicates their natural habitat, sloths may not be able to survive outside of it due to their unique genetic expression profile [58,59,60]. One potential benefit of sloth conservation is the opportunity for medical applications for humans. Previous reports have shown that sloth hair is a source of molecules active against parasites, bacteria, and cancer [61]. Our findings show a genetic basis for increased cancer resistance, telomere maintenance, and longevity in sloths. Studying the underlying genetic mechanisms behind these traits could potentially lead to both a better understanding of cancer and longevity, as well as future gene therapies targeting these factors.

Finally, we wish to address the potential limitations of this study. Due to being an exploratory pilot study, the human sample size is small with only two participants. Although imperfect, the effect of individual genetic variation was slightly controlled for by ensuring both participants were of the same sex, same ethnicity, and from the same geographic location, with the only difference being age. The same limitations apply to the sloth, of which there was only one young female subject. As such, despite general trends being in line with previous research, the results described previously may be biased in terms of age and sex and may also be influenced somewhat by individual genetic variation.

## 5. Conclusions

In conclusion, we have performed a genomic and transcriptomic analysis of *C. didactylus* in order to shed light onto the genetic basis of their unique biochemical, physiological, and behavioral characteristics. These findings are significant for two reasons. First, through comparison of the two-toed sloth and human WGS data, the shared overlap was estimated and compared to other animals. Second, through an exploratory DEG analysis between two-toed sloths and humans, several biologically relevant genes were found and tentatively linked to two-toed sloth behavior and characteristics. Although a small number of samples were collected for this initial exploratory study, which limits the generalizability of the findings, with the development of an experimental framework and standardized methodology from this study, subsequent research will control for individual genetic variation by increasing the numbers of human and sloth samples as well as expand the number of sloth species for a more generalized comparison. Potential species of interest include those that comprise the genetically distant three-toed sloths [4,5]. Overall, our findings demonstrate the first known direct genomic comparison between two-toed sloths and humans and constitute a foundation for future investigations into specific gene and protein functions tied to the unique characteristics of the sloth species.

## Figures and Tables

**Table 1:** Summary of whole genome sequencing (WGS) results for *C. didactylus* and *H. sapiens*. Compared to humans, two-toed sloths have 5 more chromosomes and a slightly longer read length. The amount of genetic overlap between two-toed sloths and humans is estimated to be ~61%. A full table can be found in the Supplementary Materials (Tables S1 and S2).

Species	Number of Chromosomes	Length (bp)	Number of Genes (Protein Coding)	Shared Orthologs
*Choloepus* *didactylus*	28	3,130,174,040	23,626	14,320
*Homo sapiens*	23	3,088,286,401	23,306	

**Table 2:** Observational data collected for two-toed sloth food intake and excreta. The values in brackets are scaled by mass (6.35 kg for the two-toed sloth, 65.4 kg for the human female). Two-toed sloths consume approximately the same number of calories as a human female but produce far more feces and far less urine.

Category	Two-Toed Sloth	Human (Female)	Difference (%) *
Caloric Intake (kcal/day) [kcal/day/kg]	228.79 [38.78]	2000 [37.74]	+3%
Feces (g/day) [g/day/kg]	33.64 [5.7]	99 [1.9]	+205%
Urine (mL/day) [mL/day/kg]	81.82 [13.87]	1400 [26.42]	−48%

* Values in the “Difference (%)” column are scaled by mass (kg).

**Table 3:** Representative table summarizing fold changes and *p*-values for shared DEGs related to metabolism. The log2 fold change threshold was 1 and the adjusted *p*-value threshold was 0.1. Values for the other 6 identified categories can be found in the Supplementary Materials (Table S3).

Category	Gene	Log2 Fold Change		Adjusted *p*-Value	
		Human A	Human B	Human A	Human B
**Metabolism**	*PKLR*	8.5	5.9	<0.001	<0.001
	*PRKAA1*	6.0	6.0	<0.001	<0.001
	*PPARGC1A*	5.2	6.3	<0.001	<0.001
	*HK1*	3.5	5.8	<0.001	<0.001
	*HK2*	2.2	3.6	<0.001	<0.001
	*PKM*	−2.0	-	<0.001	-
	*PPARA*	−3.1	−3.2	<0.001	<0.001
	*THRB*	−7.3	−6.0	<0.001	<0.001

## Data Availability

Sloth WGS data has been submitted to the NIH GenBank database. Sloth RNA data has been submitted to the NIH NCBI GEO database. Human WGS and RNA data is available upon request from the corresponding author.

## Supplementary Materials

The following supporting information can be downloaded at: https://www.mdpi.com/article/10.3390/genes16030272/s1, Figure S1: DEG heatmap for the comparison performed between the two-toed sloth and human volunteer ‘A’; Figure S2: DEG heatmap for the comparison performed between the two-toed sloth and human volunteer ‘B’; Figure S3: Transcript expression levels for four body temperature regulation-related genes between a two-toed sloth and humans; Figure S4: Transcript expression levels for eight cell cycle regulation-related genes between a two-toed sloth and humans; Figure S5: Transcript expression levels for six cancer-related genes between a two-toed sloth and humans; Figure S6: Transcript expression levels for six circadian rhythm-related genes between a two-toed sloth and humans; Figure S7: Transcript expression levels for four core circadian rhythm-related genes between a two-toed sloth and humans; Figure S8: Transcript expression levels for five telomere maintenance-related genes between a two-toed sloth and humans; Figure S9: Transcript expression levels for six longevity-related genes between a two-toed sloth and humans; Table S1: Summary of whole genome sequencing (WGS) data for human (female) chromosomes; Table S2: Summary of whole genome sequencing (WGS) data for Linnaeus’s two-toed sloth (female) chromosomes; Table S3: Table summarizing fold changes and *p*-values for shared DEGs related to body temperature, cell cycle, cancer, circadian rhythms, telomere maintenance, and longevity. The log2 fold change threshold was 1 and the adjusted *p*-value threshold was 0.1.
