# Supplementary material for: An Exploratory Genomic and Transcriptomic Analysis Between Choloepus didactylus and Homo sapiens
Source: Genes (Basel). 2025 Feb 25;16(3):272. doi: 10.3390/genes16030272 (PMC11942560; doi:10.3390/genes16030272)
Supplement: Supplementary file 1 [file genes-16-00272-s001.zip › genes-3477458-supplementary.pdf]

Supplementary Information For:

An Exploratory Genomic and Transcriptomic Analysis Between *Choloepus didactylus* and *Homo sapiens*

Figures and Tables

Table S1: Summary of whole genome sequencing (WGS) data for human (female) chromosomes.

Chromosome (Human)	Length (bp)	Number of Genes (Protein-Coding)	Number of Shared Orthologs in Two-Toed Sloth Genome	Corresponding Chromosome(s) in Two-Toed Sloth
1	248,956,422	2,220	1,525	Chr 2<br>Chr 11<br>Chr 24
2	242,193,529	1,321	998	Chr 20<br>Chr 17<br>Chr 9
3	198,295,559	1,119	885	Chr 1
4	190,214,555	798	592	Chr 3
5	181,538,259	1,005	671	Chr 11<br>Chr 13<br>Chr 24
6	170,805,979	2,046	667	Chr 7<br>Chr 2<br>Chr 24
7	159,345,973	1,006	689	Chr 21<br>Chr 5
8	145,138,636	861	481	Chr 20<br>Chr 3

				Chr 14
9	138,394,717	807	572	Chr 10
10	133,797,422	752	584	Chr 5<br>Chr 8<br>Chr 15
11	135,086,622	1,432	840	Chr 6
12	133,275,309	1,100	801	Chr 8<br>Chr 23
13	114,364,328	349	268	Chr 12<br>Chr 17
14	107,043,718	691	449	Chr 4
15	101,991,189	766	450	Chr 4
16	90,338,345	947	572	Chr 21<br>Chr 22
17	83,257,441	1,411	795	Chr 18
18	80,373,285	277	223	Chr 16
19	58,617,616	2,094	873	Chr 25<br>Chr 27
20	64,444,167	553	432	Chr 19
21	46,709,983	258	122	Chr 1
22	50,818,468	494	323	Chr 8<br>Chr 23
X	156,040,895	897	493	Chr 24<br>Chr X<br>Chr Y
Y	57,227,415	89	2	Chr X
MT	16,569	13	13	Chr MT
**Total**	**3,088,286,401**	**23,306**	**14,320**	**Chr 1-25**<br>**Chr 27**<br>**Chr X**<br>**Chr Y**<br>**Chr MT**

Table S2: Summary of whole genome sequencing (WGS) data for Linnaeus's two-toed sloth (female) chromosomes.

Chromosome (Sloth)	Length (bp)	Number of Genes (Protein Coding)	Number of Shared Orthologs in Human Genome	Corresponding Chromosome(s) in Human
1	250,664,781	1,515	1,009	Chr 21<br>Chr 3
2	248,084,734	2,239	1,555	Chr 6<br>Chr 1
3	223,233,325	980	646	Chr 4<br>Chr 8
4	196,373,313	1,474	901	Chr 14<br>Chr 15
5	168,700,187	894	565	Chr 10<br>Chr 7
6	156,663,619	1,844	838	Chr 11
7	156,022,981	1,104	584	Chr 6
8	146,178,362	1,331	861	Chr 22<br>Chr 12<br>Chr 10
9	135,328,281	742	500	Chr 2
10	132,469,143	965	571	Chr 9
11	90,811,022	553	283	Chr 5<br>Chr 1
12	102,976,318	405	263	Chr 13
13	100,229,138	603	385	Chr 5
14	100,080,887	511	318	Chr 8
15	89,644,403	660	462	Chr 10
16	86,887,137	362	223	Chr 18

17	78,810,110	630	359	Chr 2<br>Chr 13
18	77,031,504	1,245	795	Chr 17
19	66,415,491	603	430	Chr 20
20	64,556,003	385	254	Chr 2<br>Chr 8
21	53,136,999	776	483	Chr 16<br>Chr 7
22	44,682,675	401	288	Chr 16
23	32,044,896	459	312	Chr 12<br>Chr 22
24	25,766,288	253	71	Chr 5<br>Chr 1<br>Chr 6<br>Chr X
25	10,490,878	680	400	Chr 19
26	17,351,548	80	0	NA
27	26,625,015	770	472	Chr 19
X	193,839,925	909	404	Chr X<br>Chr Y
Y	55,058,534	240	75	Chr X
MT	16,543	13	13	Chr MT
**Total**	**3,130,174,040**	**23,626**	**14,320**	**Chr 1-22**<br>**Chr X**<br>**Chr Y**<br>**Chr MT**

Table S3: Table summarizing fold changes and p-values for shared DEGs related to body temperature, cell cycle, cancer, circadian rhythm, telomere maintenance, and longevity. The log2 fold change threshold was 1 and the adjusted p-value threshold was 0.1.

Category	Gene	log2 Fold Change		Adjusted p-Value	
		Human A	Human B	Human A	Human B
**Body Temperature**	PTGES	4.8	2.1	<0.001	<0.001
	PTGS2	4.6	3.2	0.002	0.008
	PTGES2	-2.7	-2.9	<0.001	<0.001
	THRB	-7.3	-6.0	<0.001	<0.001
**Cell Cycle**	E2F2	9.8	-	<0.001	-
	CCND1	4.4	6.8	<0.001	<0.001
	CCNA1	2.2	-	0.005	-
	E2F7	1.5	2.6	0.001	<0.001
	E2F1	-3.9	-	<0.001	-
	CDKN1A	-	2.5	-	<0.001
	E2F8	-	1.8	-	<0.001
	MCM2	-	1.6	-	<0.001
**Cancer**	BRCA2	6.8	8.4	<0.001	<0.001
	NF2	4.1	2.6	0.001	<0.001
	BRCA1	2.5	1.9	<0.001	<0.001
	TP53	1.9	-	<0.001	-
	NF1	1.6	1.9	<0.001	<0.001
	APC	1.6	-	<0.001	-
**Circadian Rhythm**	GABRA1	3.2	2.8	0.06	0.08
	ALDH7A1	2.8	3.5	<0.001	<0.001
	HDC	2.0	-4.9	<0.001	<0.001
	MAOB	-2.2	1.6	<0.001	<0.001
	PER3	3.3	4.1	<0.001	<0.001
	PER1	3.1	2.7	<0.001	<0.001
	CLOCK	-0.3	-0.6	0.03	<0.001
	CRY1	1.2	1.0	<0.001	<0.001
	CRY2	-0.4	-1.1	<0.001	<0.001

	PER2	-0.3	-0.2	0.25	0.18
**Telomere Maintenance**	TERT	3.2	-	0.007	
	RTEL1	3.2	3.4	<0.001	<0.001
	STN1	2.2	-	0.001	
	WRAP53	2.2	1.5	<0.001	<0.001
	POT1	1.7	1.6	<0.001	<0.001
**Longevity**	UCP2	3.9	4.8	<0.001	<0.001
	MTOR	2.6	-	<0.001	-
	TP53	1.9	-	<0.001	-
	IGF1	-	4.6	-	<0.001
	APOE	-	2.1	-	<0.001
	KL	-	-1.9	-	0.07

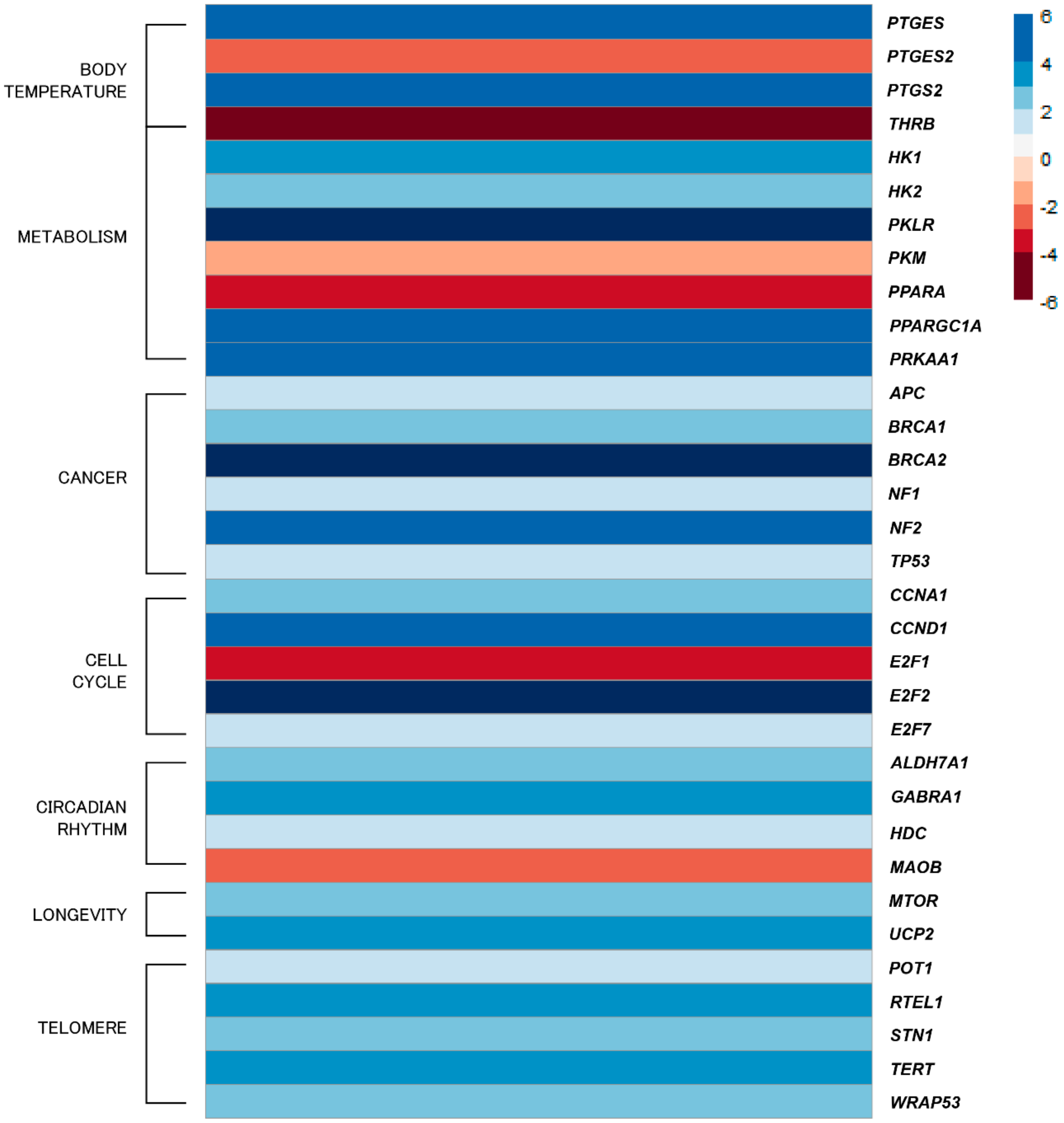

Figure S1: DEG heatmap for the comparison performed between the two-toed sloth and human volunteer ‘A’.

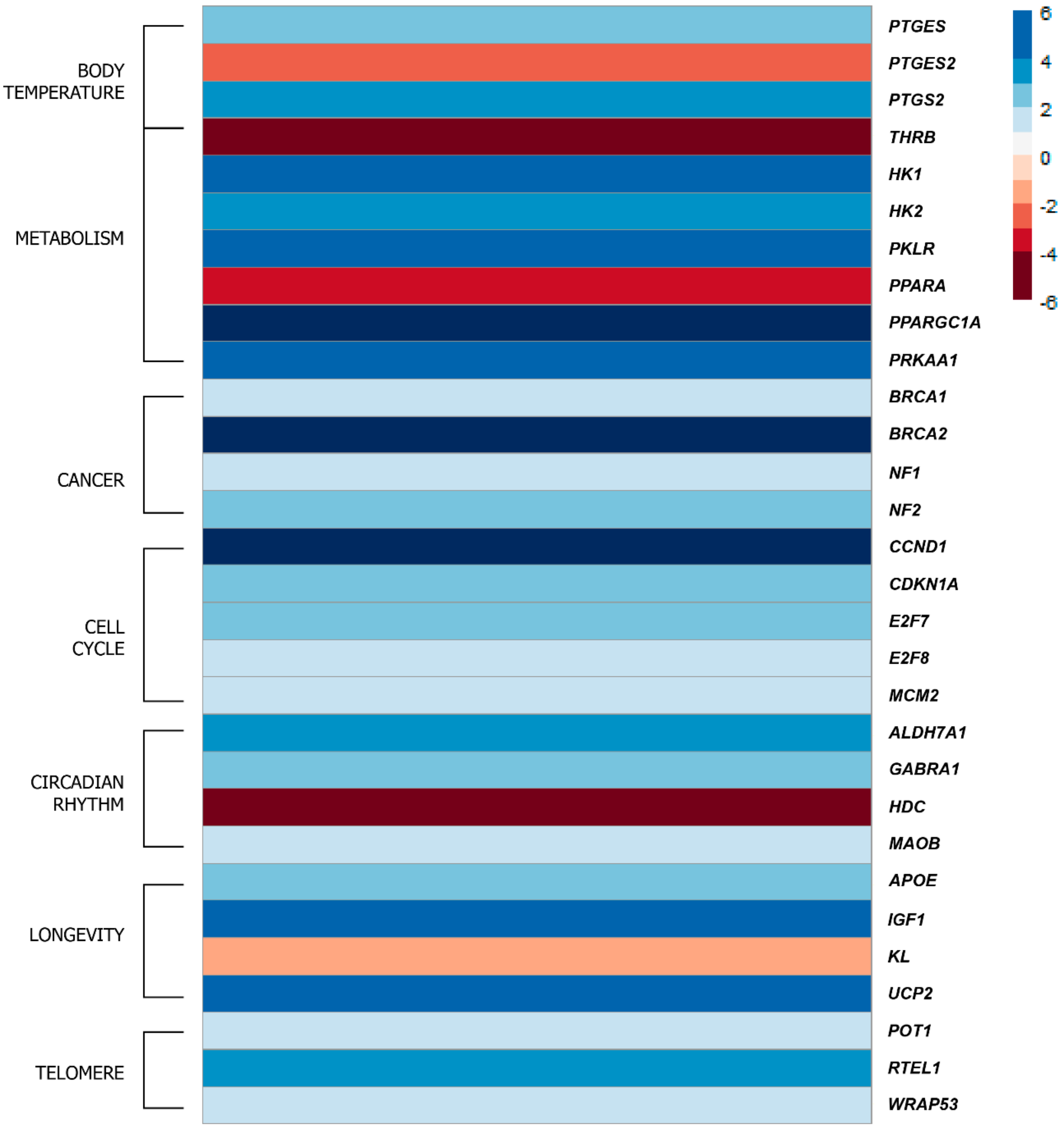

Figure S2: DEG heatmap for the comparison performed between the two-toed sloth and human volunteer 'B'.

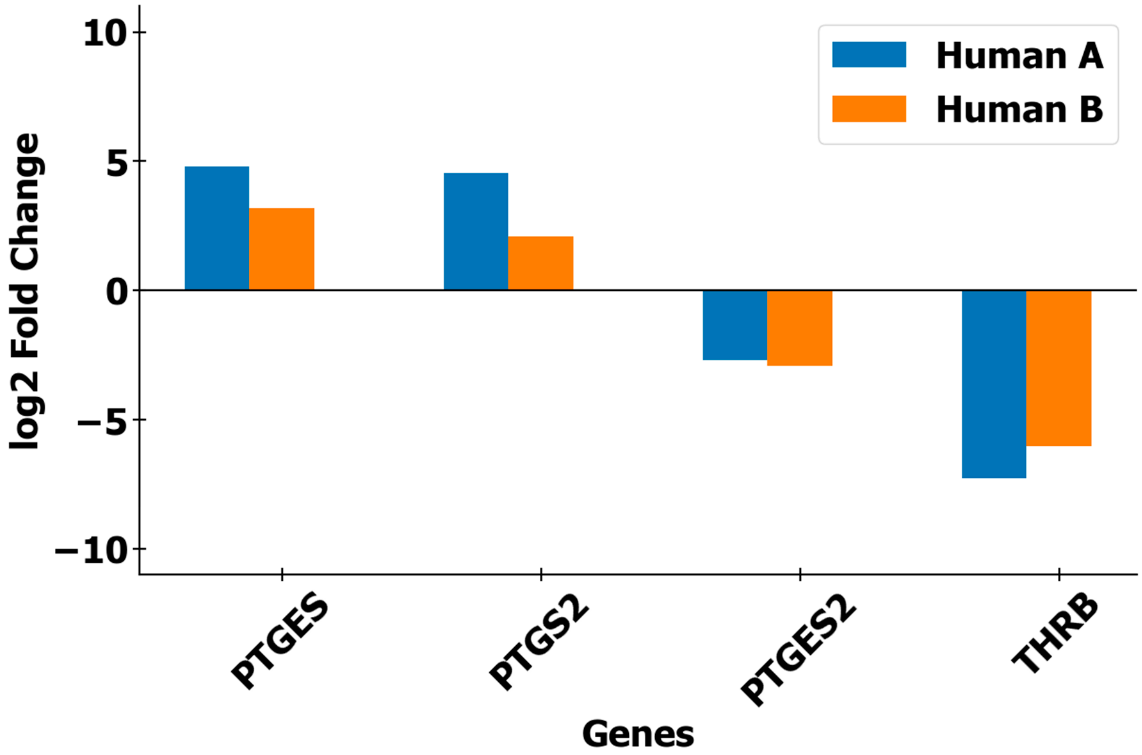

Figure S3: Transcript expression levels for 4 body temperature regulation-related genes between a two-toed sloth and humans. The blue bars represent two-toed sloth expression levels relative to human volunteer 'A' while the orange bars represent two-toed sloth expression levels relative to human volunteer 'B'.

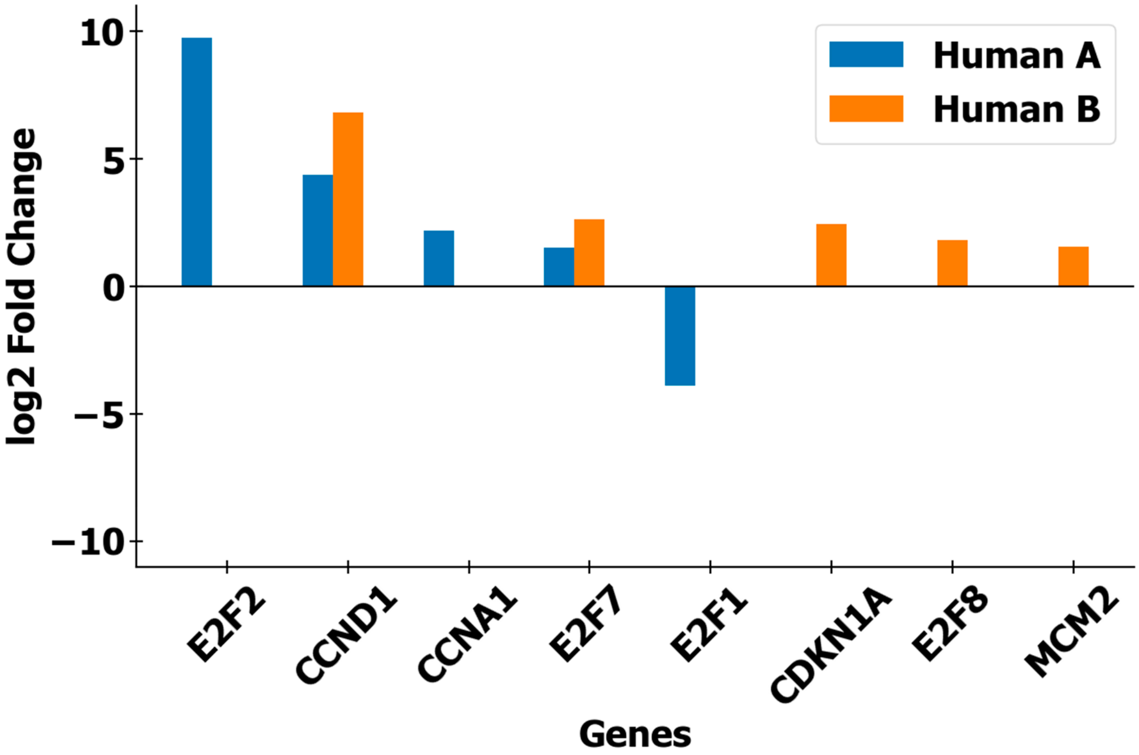

Figure S4: Transcript expression levels for 8 cell cycle regulation-related genes between a two-toed sloth and humans. The blue bars represent two-toed sloth expression levels relative to human volunteer 'A' while the orange bars represent two-toed sloth expression levels relative to human volunteer 'B'.

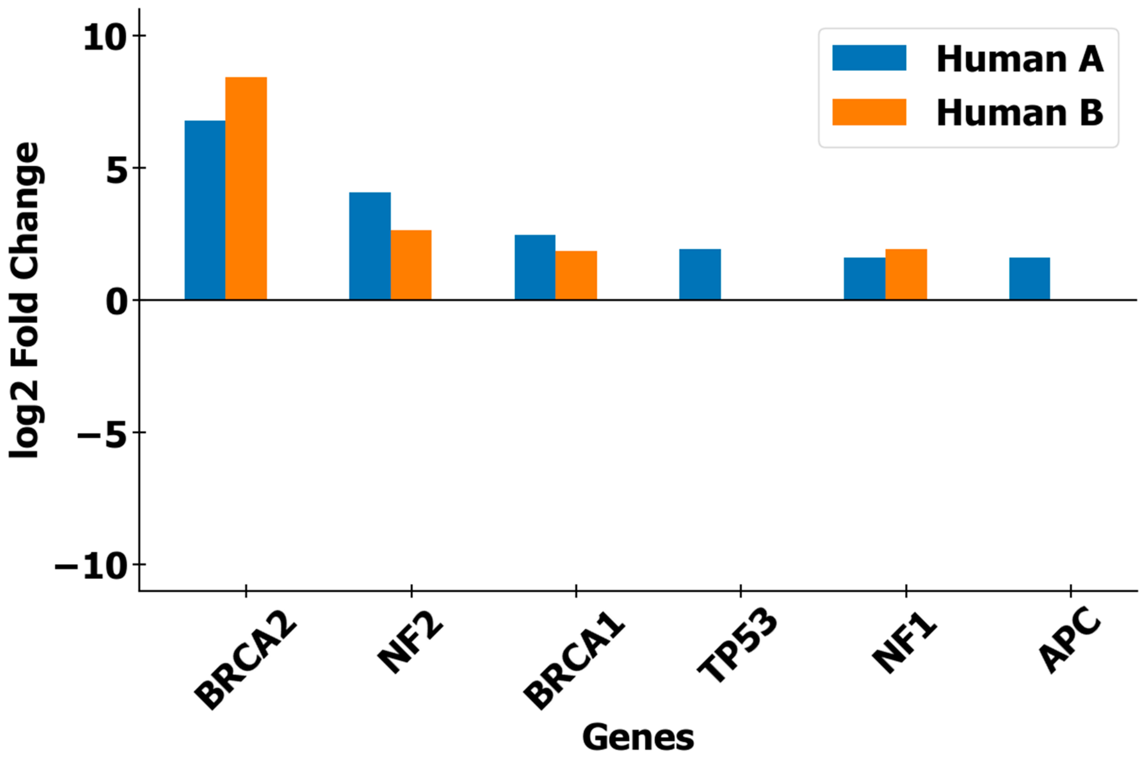

Figure S5: Transcript expression levels for 6 cancer-related genes between a two-toed sloth and humans. The blue bars represent two-toed sloth expression levels relative to human volunteer 'A' while the orange bars represent two-toed sloth expression levels relative to human volunteer 'B'.

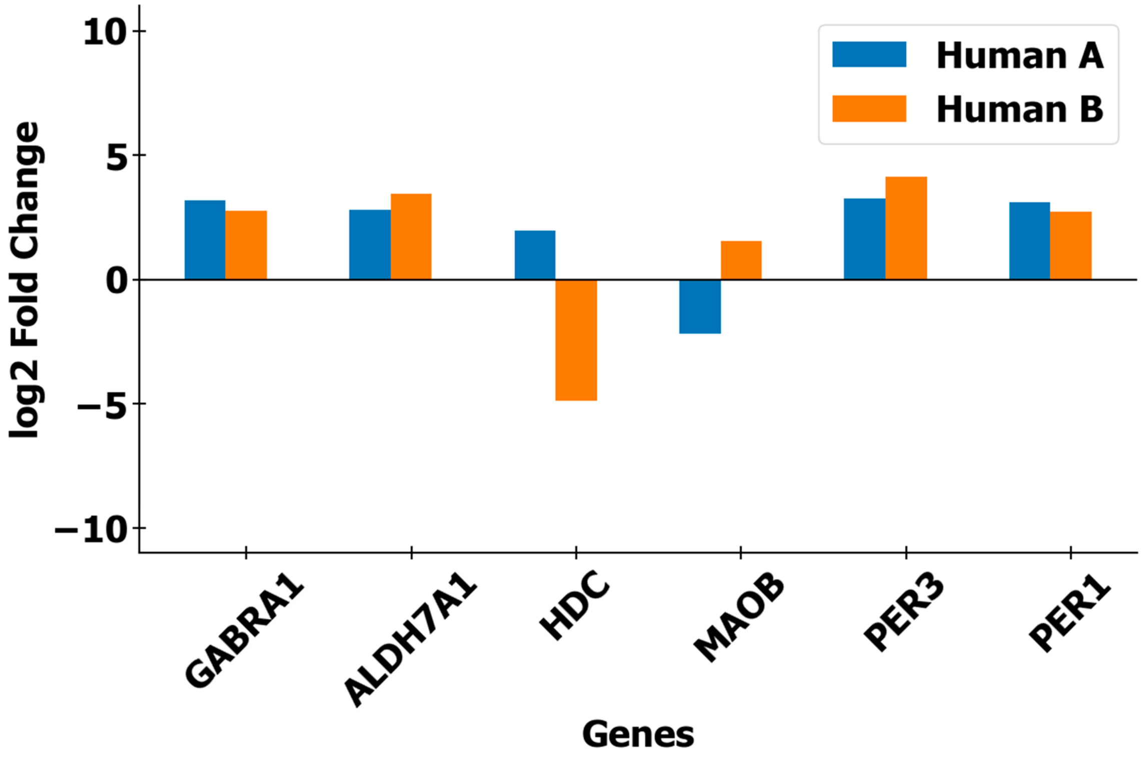

Figure S6: Transcript expression levels for 6 circadian rhythm-related genes between a two-toed sloth and humans. The blue bars represent two-toed sloth expression levels relative to human volunteer ‘A’ while the orange bars represent two-toed sloth expression levels relative to human volunteer ‘B’.

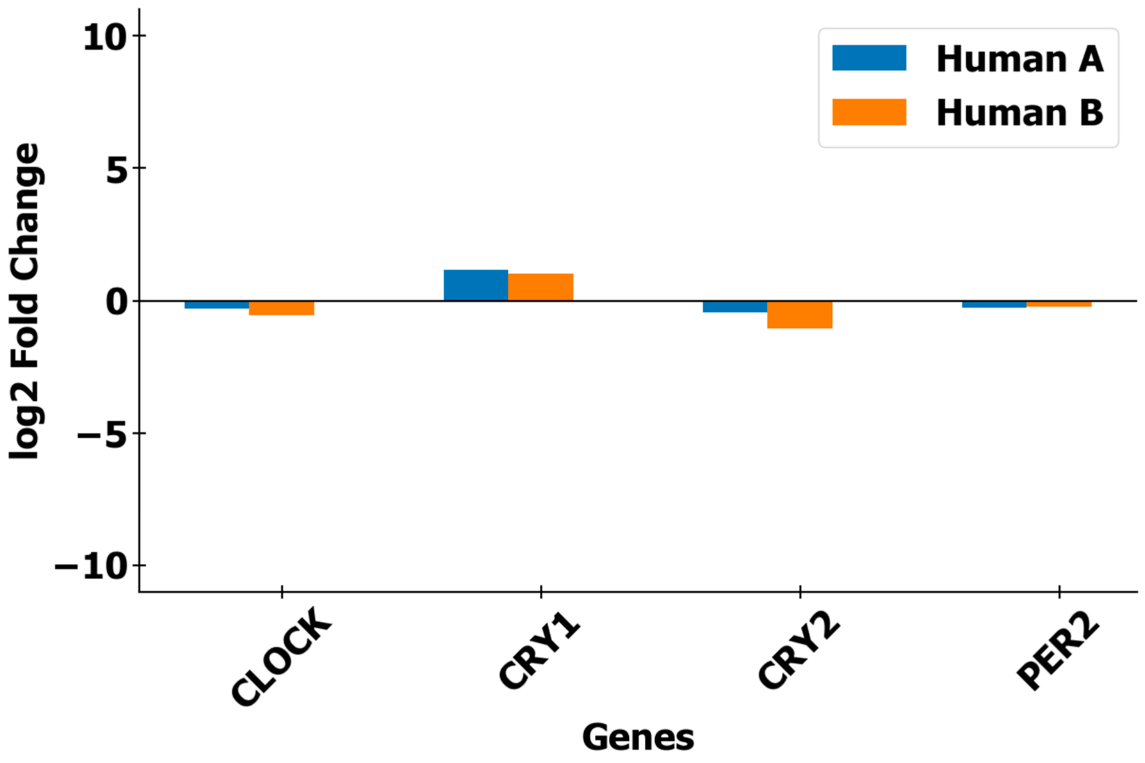

Figure S7: Transcript expression levels for 4 core circadian rhythm-related genes between a two-toed sloth and humans. The blue bars represent two-toed sloth expression levels relative to human volunteer 'A' while the orange bars represent two-toed sloth expression levels relative to human volunteer 'B'. Due to the low fold changes (<1), these displayed genes were not considered 'differentially expressed'.

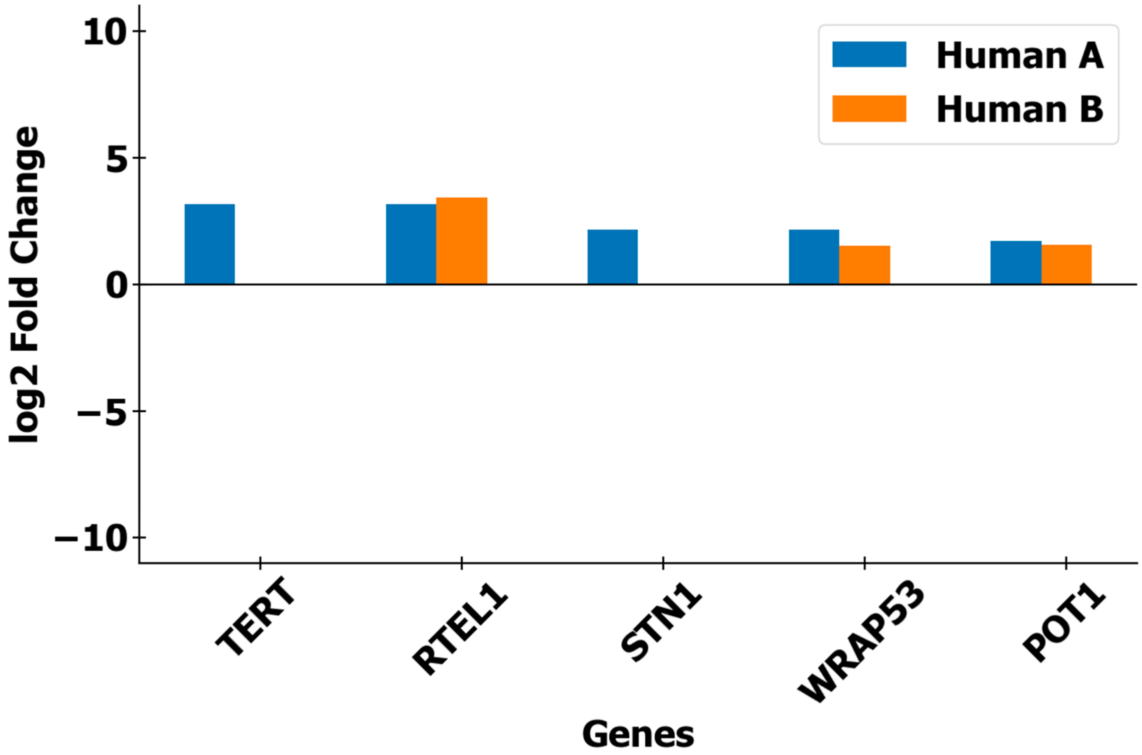

Figure S8: Transcript expression levels for 5 telomere maintenance-related genes between a two-toed sloth and humans. The blue bars represent two-toed sloth expression levels relative to human volunteer ‘A’ while the orange bars represent two-toed sloth expression levels relative to human volunteer ‘B’.

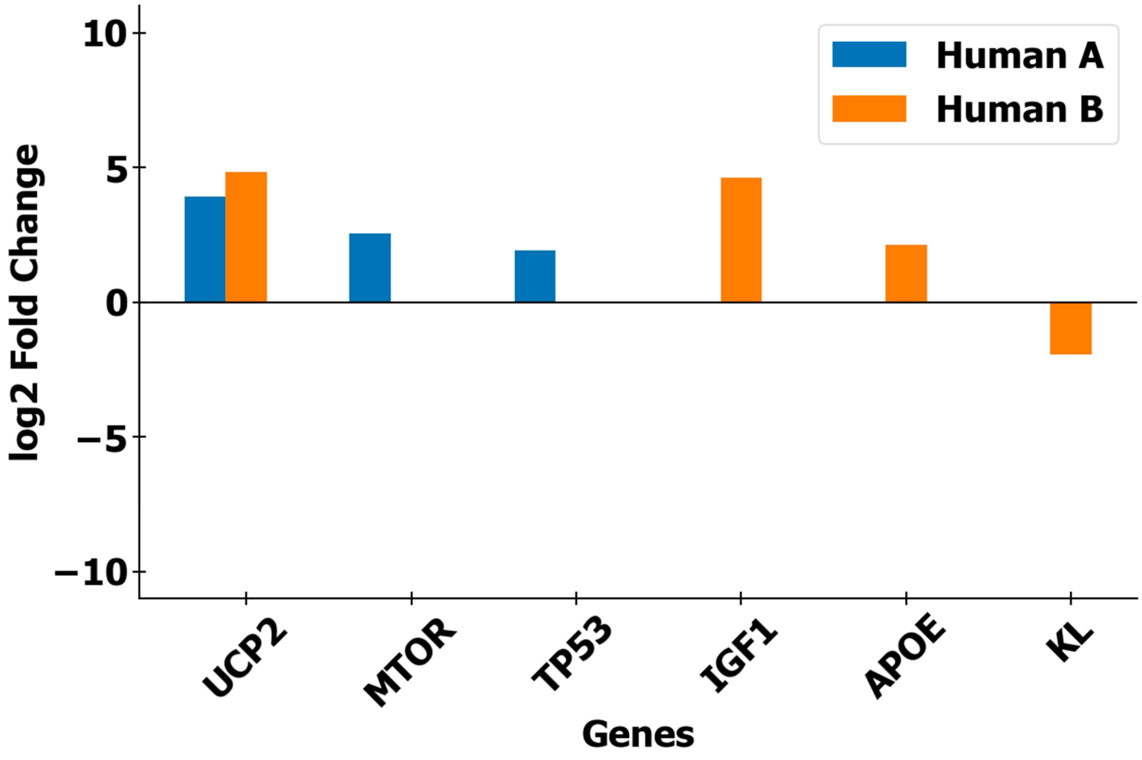

Figure S9: Transcript expression levels for 6 longevity-related genes between a two-toed sloth and humans. The blue bars represent two-toed sloth expression levels relative to human volunteer ‘A’ while the orange bars represent two-toed sloth expression levels relative to human volunteer ‘B’.
